# Hydroxychloroquine and Cardiovascular Events in Patients With Systemic Lupus Erythematosus

**DOI:** 10.1001/jamanetworkopen.2024.32190

**Published:** 2024-08-30

**Authors:** Lamiae Grimaldi, Tom Duchemin, Yann Hamon, Albert Buchard, Jacques Benichou, Lucien Abenhaim, Nathalie Costedoat-Chalumeau, Yola Moride

**Affiliations:** 1 Department of Pharmacology, Hospital Group Paris-Saclay, Assistance Publique-Hôpitaux de Paris, Paris, France; 2 Centre for Epidemiology and Population Health, Team Anti-infective evasion and Pharmacoepidemiology, Inserm U1018, Villejuif, France; 3 Department of Pharmacoepidemiology, Faculty of medicine and health science, University Versailles Saint-Quentin/Paris-Saclay, Paris, France; 4 RE-MEDs, France, Rueil Malmaison, France; 5 Department of Biostatistics, Centre Hospitalier Universitaire, CHU Rouen, Rouen, France; 6 Centre for Epidemiology and Population Health, Team High-dimensional Biostatistics, Inserm U1018, Villejuif, France; 7 RE-MEDs, London, United Kingdom; 8 London School of Hygiene and Tropical Medicine, London, United Kingdom; 9 Department of Internal Medicine, Cochin Hospital, Centre de référence maladies auto-immunes et systémiques rares d’île de France, Assistance Publique-Hôpitaux de Paris, Paris, France; 10 University of Paris, Centre for Epidemiology and Biostatistics Research of Sorbonne Paris Cité, Paris, France; 11 Rutgers, The State University of New Jersey, New Brunswick

## Abstract

**Importance:**

Systemic lupus erythematosus (SLE) predisposes individuals to early cardiovascular (CV) events. While hydroxychloroquine is thought to mitigate CV risk factors, its protective role against CV events, particularly arterial ones, remains to be confirmed.

**Objective:**

To evaluate the association between hydroxychloroquine and the risk of myocardial infarction (MI), stroke, and other thromboembolic events (OTEs) in patients with SLE.

**Design, Setting, and Participants:**

This cohort study using a nested case-control design was conducted within the National French Healthcare Database (SNDS), which represents 99% of the French population, from 2010 to 2020. Participants were the cohort of all patients with SLE recorded in the SNDS. Patients with SLE experiencing CV events during the study period were the case group; those without CV events were controls. The analysis period was from February 2022 to September 2023.

**Exposures:**

Hydroxychloroquine use within 365 days prior to the index date, defined as current (within 90 days), remote (91-365 days), or no exposure within the previous 365 days.

**Main Outcomes and Measures:**

Outcomes of interest were MI, stroke, and OTE, analyzed individually and as a composite outcome (primary analysis). Controls were matched to patients with CV events by age, sex, time since SLE onset and entry into the SNDS database, index date, prior antithrombotic and CV medication, chronic kidney disease, and hospitalization. Multivariable conditional logistic regression was performed using hydroxychloroquine exposure as the main independent variable.

**Results:**

The SLE cohort included 52 883 patients (mean [SD] age, 44.23 [16.09] years; 45 255 [86.6%] female; mean [SD] follow-up, 9.01 [2.51] years), including 1981 patients with eligible CV events and 16 892 matched control patients. There were 669 MI events, 916 stroke events, and 696 OTEs in the individual outcome studies. For current exposure to hydroxychloroquine, the adjusted odds were lower for composite CV events (odds ratio [OR], 0.63; 95% CI, 0.57-0.69) as well as for MI (OR, 0.72; 95% CI, 0.60-0.85), stroke (OR, 0.69; 95% CI, 0.60-0.81), and OTEs (OR, 0.58; 95% CI, 0.49-0.69) individually compared with no hydroxychloroquine exposure within 365 days.

**Conclusions and Relevance:**

In this nationwide cohort study of patients with SLE, a protective association was found between the current use of hydroxychloroquine and the occurrence of CV events, but not between remote use of hydroxychloroquine and CV outcomes, highlighting the value of continuous hydroxychloroquine treatment in patients with SLE.

## Key Points

**Question:**

What is the association of hydroxychloroquine with the overall risk of cardiovascular (CV) events in patients with systemic lupus erythematosus (SLE)?

**Findings:**

In this cohort study among 52 883 patients with SLE, 2107 patients with composite CV outcomes (myocardial infarction, stroke, and other thromboembolic events) and 16 892 matched controls were identified. Compared with no hydroxychloroquine exposure within 365 days, patients with current hydroxychloroquine exposure had significantly lower odds of the composite CV outcome and of myocardial infarction, stroke, or 0.58 for other thromboembolic events individually.

**Meaning:**

Continued use of hydroxychloroquine was associated with a lower risk of myocardial infarction, stroke, and other thromboembolic events.

## Teaser

This cohort study evaluates the association of current, recent, and no hydroxychloroquine use with the risk of myocardial infarction, stroke, and other thromboembolic events in patients with systemic lupus erythematosus.

## Introduction

Systemic lupus erythematosus (SLE) is a chronic systemic autoimmune disease characterized by flare-ups and organ damage.^1,2,3,4^ Recent studies have demonstrated that patients with SLE treated with hydroxychloroquine had a better overall relative survival rate than untreated patients,^5^ and that they were significantly less likely to develop severe SLE, while discontinuing hydroxychloroquine was associated with increased risk of disease flare-ups in the months after discontinuation.^5,6,7^ In parallel, recent studies^8,9^ identified trends in the reduction of myocardial infarction (MI), stroke, and venous thromboembolism among patients with SLE, but lacked statistical power to conclude a significant benefit associated with hydroxychloroquine use.^8^ However, data on the protective effects of hydroxychloroquine on individual cardiovascular (CV) events, especially arterial events, in patients with SLE, remain scarce.

We aimed to evaluate the association between current hydroxychloroquine exposure and the overall relative risk of composite CV events and individual events (MI, stroke, and other thromboembolic events [OTEs]) in patients with SLE. Evaluating the persistence of this link after discontinuation of hydroxychloroquine was the secondary objective.

## Methods

This cohort study was approved by the French Data Protection Supervisory Authority (Commission Nationale de l’Informatique et des Libertés), which also determined that since the data were anonymous, no informed consent was required. This study is reported following the Strengthening the Reporting of Observational Studies in Epidemiology (STROBE) reporting guideline.

### Overview of the Study Design

This study used a nested case-control design of CV events and exposure to hydroxychloroquine among adult patients with SLE identified in the National System of Health Databases (SNDS) from 2010 to 2020.^10^ The analysis period was from February 2022 to September 2023.

### Data Source

The SNDS covers 99% of the national residents of France (approximately 66 million individuals) from birth or immigration to the country to emigration or death, making this electronic health data source one of the largest and most comprehensive health care databases used for pharmacoepidemiology studies.^11^ The SNDS links health care data (billing for all medical services, drug dispensations in ambulatory pharmacies, laboratory tests, and procedures) with the National Hospital Discharge Database (principal, related, and associated diagnoses of all hospitalizations, including day-hospitalization and emergency department visits, either in public or private hospitals, coded in the *International Statistical Classification of Diseases and Related Health Problems, Tenth Revision *[*ICD-10*]).^11^ Every French resident is covered by one or another public health insurance, and all data are consolidated in the SNDS. All drugs reported in this study are covered by the national health insurance. A unique feature of this database is a variable indicating patients receiving universal coverage for all health care–related expenses for any long-term condition (affection of long duration [ALD]), such as SLE, recorded alongside the date of disease onset.

### Populations

The cohort of adults aged at least 18 years with SLE was identified within the SNDS registry using the following algorithm: either (1) the patient met at least 2 of the following criteria: 1 hospitalization with a discharge diagnosis of SLE, identified through *ICD-10* coding, at least 1 dispensing record for a drug prescribed to treat SLE identified through the Anatomical Therapeutic Chemical (ATC) codes (including mycophenolate mofetil, azathioprine, hydroxychloroquine, thalidomide, methotrexate, belimumab, and cyclophosphamide), or an active ALD with a recorded SLE *ICD-10* code (M32) or (2) the patient had at least 2 hospitalizations during the study period (2010-2020) using a recorded *ICD-10* diagnostic code for SLE (≥1 of which had to be recorded as a principal diagnosis). All codes used are reported in eTable 1 in Supplement 1.

Entry into the SLE cohort was defined as recording the first SLE-related code in the SNDS database. Patients with SLE were followed-up until the end of the study period (December 31, 2020), exit from the database, or death, whichever occurred first.

### CV Event Definitions

The study outcomes were a composite of the following CV events and each CV event individually: MI, stroke (including transient ischemic attack), and OTEs (eg, phlebitis and thrombophlebitis, other venous thrombosis, venous thromboembolism, and pulmonary embolism). Events were identified using the *ICD-10* codes reported in eTable 1 in Supplement 1. For each outcome (composite or individual), the index date was the first record of a diagnostic code for the aforementioned events after entry into the SLE cohort.

The case group was patients with a first CV outcome event occurring at least 5 years after entry into the database to guarantee a sufficiently long period without CV outcomes. A secondary analysis allowed that period to be reduced to 2 years. In both analyses, patients also were required to have at least 3 months after entry into the SLE cohort to ensure a reasonably long window of opportunity for hydroxychloroquine treatment. A sensitivity analysis used only 2 years without any CV outcome events after entry into the study.

### Controls

Up to 10 controls per patient with a CV event were identified using incidence density sampling^12^ of all patients present in the SLE cohort at the time of the index date (±1 year) of their matched patient with a CV event who had not experienced a CV event by that date. The pool of controls consisted of risk sets of SLE cohort members without history of CV outcome events at the time of the index date in the matched case group. Accordingly, patients who eventually experienced a CV event could serve as controls until their first CV outcome event. Patients with CV events and controls were matched on age (±1 year), sex, time since the first lupus ALD code (M32) in the database (±10% if the time was <1 year, ±40% otherwise), time since entry in the SNDS database (±30 days), index date (±1 year), use in the past 365 days before the index date of platelet aggregation inhibitor drugs (identified with ATC code beginning with B01AC), use of CV drugs (identified with an ATC code beginning with C), previous chronic kidney disease (CKD) diagnosis (*ICD-10* code N18 or dialysis), and at least 1 hospitalization in the last 365 days. The definitions and codes of these variables are available in eTable 2 in Supplement 1.

### Assessment of Exposure

Exposure was ascertained by pharmacy dispensation of hydroxychloroquine during the 365 days before the index date. In France, hydroxychloroquine is dispensed in packages containing 30 pills of 200 mg of the active ingredient. A dispensation of 1 package of hydroxychloroquine was considered equivalent to 30 days of drug exposure; the same-day dispensation of 2 packages of hydroxychloroquine was considered a 60-day exposure. Overlapping times between consecutive dispensations were subtracted. Hydroxychloroquine exposure was categorized as current exposure whenever the last day of estimated exposure occurred on the index date or within 90 days before the index date, remote exposure when it occurred between 91 and 365 days before the index date, or no exposure within 365 days when no estimated exposure occurred in the 365 days preceding the index date. All exposure categories were mutually exclusive. Additionally, a category labeled as past use of hydroxychloroquine was established, indicating instances where hydroxychloroquine exposure occurred within the 4 years preceding the 365 days before the index date but not during this specific 365-day period.

### Covariates

In addition to matching on age, sex, duration of disease, CV treatments, antiplatelet treatment, CKD, and hospitalization in the past year, a number of covariates were assessed within the 365 days before index date to minimize sources of confounding. These included age (as a continuous variable), other CV events (for individual event analysis), use of nonsteroidal anti-inflammatory drugs, glucocorticoids, SLE treatments other than hydroxychloroquine, and the number of medical visits for any cause. Acute nephritis, cancer, diabetes, and antiphospholipid syndrome (APLS) were considered as additional covariates. The codes of the covariates are presented in eTable 3 in Supplement 1.

### Statistical Analysis

Means and SDs were used to describe continuous variables expected to be normally distributed, while proportions were used for categorical data. Descriptive analyses of composite CV outcomes and matched controls were performed according to CV risk factors (matching criteria and covariates) and hydroxychloroquine exposure.

Crude-matched and multivariable conditional logistic regression analyses were performed to estimate odds ratios (ORs) and 95% CIs for the events of interest, using no exposure in the past 365 days as the reference. Multivariable analyses were adjusted for the aforementioned covariates. Sensitivity analyses (controlling for additional covariates, stratifying into age groups, according to the use of CV drugs, by a prior history of CV events, or the presence of APLS) were also performed. A sensitivity analysis controlling for past exposure to hydroxychloroquine was also conducted.^13^

Statistical analyses were performed using SAS Enterprise Guide software version 7.15 (SAS Institute). Statistical significance was set at *P* < .05, and all *P* values were 2-sided.

## Results

Of 52 883 adult patients with SLE (mean [SD] age, 44.23 [16.09] years; 45 255 [86.6%] female; mean [SD] follow-up, 9.01 [2.51] years) included in the cohort, 1981 patients with a CV event could be matched with 16 892 controls (mean ratio, 1:8.5).

Among patients with SLE cohort, 4210 patients experienced a CV event, with 2107 (52%) occurring 5 years after entry in the registry and 3 months after first SLE diagnosis. When the delay was reduced to 2 years, this number increased to 2922 (69.4%). Specifically, there were 623 MI events, 865 stroke events, and 696 OTE events (Table 1).

**Table 1.** Patients With CV Events Identified in the Cohort of Patients With Systemic Lupus Erythematosus During the Study Period (2010-2020)

Outcome	No.		
	Events	Eligible cases	Patients matched to controls
All CV events	4210	2107	1981
Myocardial infarction	1065	669	623
Stroke or transient ischemic attack	1752	916	865
Thromboembolism	1619	696	664
Pulmonary embolism	682	264	249
Venous thromboembolism	1017	470	450

Abbreviation: CV, cardiovascular.

At the first CV event, the mean (SD) time since SLE onset was 13.13 (7.66) years for patients with a CV event and 11.73 (7.15) years for controls (Table 2). Regarding matching criteria, more than half of the sample (1138 patients [57.4%]) had at least 1 hospitalization in the past year, and 268 patients (13.5%) had CKD. Most patients (1259 patients [63.6%]) had used a CV drug before the index date, and 573 patients (28.9%) had used antiplatelet drugs. Acute nephritis was present in 484 patients with a CV event (24.4%) and 3789 controls (22.4%), APLS in 180 patients with a CV event (9.1%) and 1161 controls (6.9%), cancer in 228 patients with a CV event (11.5%) and 1897 controls (11.2%), and diabetes in 381 patients with a CV event (19.2%) and 2595 controls (15.4%). Further descriptive statistics are presented in eTable 4 in Supplement 1.

**Table 2.** Characteristics of Individuals With SLE Matched With Controls

Characteristic	Individuals, No. (%)	
	Patients with CV events (n = 1981)	Controls (n = 16 892)
Matching variables		
Age, mean (SD), y	57.96 (16.41)	57.96 (16.41)
Time since ALD (onset of SLE), mean (SD), y	13.13 (7.66)	11.73 (7.15)
Sex		
Female	1605 (81.0)	13 686 (81.0)
Male	376 (19.0)	3206 (19.0)
Chronic kidney disease^a^	268 (13.5)	2285 (13.5)
≥1 Hospitalization^a^	1138 (57.4)	9704 (57.4)
Antiplatelet drugs^a^	573 (28.9)	4886 (28.9)
Cardiovascular drugs^a^	1259 (63.6)	10 736 (63.6)
Covariates included in the multivariate model		
Glucocorticoids^a^	1257 (63.5)	9712 (57.5)
SLE treatments (other than HCQ)^a^	509 (25.7)	4265 (25.2)
Nonsteroidal anti-inflammatory drugs^a^	662 (33.4)	5940 (35.2)
Medical visits, mean (SD), No.^a^	11.85 (13.98)	10.83 (11.93)
Acute nephritis	484 (24.4)	3789 (22.4)
Antiphospholipid syndrome	180 (9.1)	1161 (6.9)
Cancer	228 (11.5)	1897 (11.2)
Diabetes	381 (19.2)	2595 (15.4)

Abbreviations: ALD, affection of long duration; CV, cardiovascular; HCQ, hydroxychloroquine; SLE, systemic lupus erythematosus.

^a^ Assessed 365 days prior to the index date.

The 126 patients with a CV event who did not have matched controls and were excluded from further analysis were older (mean [SD] age, 66.41 [20.06] years) and more likely to be men (61 [48.4%] male) than the retained patients with CV events. This reflects the difficulties in finding older male SLE controls without previous cardiovascular events.

### Hydroxychloroquine Use

Among patients with SLE, 49 597 (87.7%) received at least 1 hydroxychloroquine dispensation during follow-up. Current users of hydroxychloroquine had a mean (SD) age of 55.48 (11.21) years compared with 60.08 (15.45) years for nonusers (Table 3). The mean (SD) time since the onset of SLE was 11.21 (6.88) years for current hydroxychloroquine users vs 7.06 (7.06) years for nonusers. Among current hydroxychloroquine users, 701 (7.6%) had CKD, compared with 734 (11.0%) among nonusers. Similarly, the prevalence of acute nephritis was 1589 patients (17.1%) vs 1269 patients (19.1%), APLS was 674 patients (7.3%) vs 390 patients (5.9%), cancer was 745 patients (8.0%) vs 848 patients (12.7%), and diabetes was 1692 patients (25.8%) and 1723 patients (18.2%) among hydroxychloroquine users and vs non-users, respectively. Regarding medication use, 2447 hydroxychloroquine users (26.4%) were using antiplatelet agents, compared with 1783 nonusers (26.8%), and 5563 hydroxychloroquine users (60.0%) were using CV drugs, compared with 4377 nonusers (65.7%). Matching of patients with CV events and controls resulted in balanced groups (Table 2).

**Table 3.** Past Hydroxychloroquine Exposure Among Controls Identified for the Study of Composite CV Events

Characteristic	HCQ exposure within 365 d prior to the index date, No. (%)		
	Current exposure (0-90 d) (n = 9274)	Remote exposure (91-365 d) (n = 955)	No exposure (0-365 d) (n = 6663)
Matching variables			
Age, mean (SD), y	55.48 (11.21)	55.21 (16.46)	60.08 (15.45)
Time since ALD [onset of illness], mean (SD), y	11.21 (6.88)	10.58 (6.55)	7.06 (7.06)
Sex			
Female	7895 (85.1)	800 (83.8)	5687 (85.4)
Male	1379 (14.9)	155 (16.2)	976 (14.6)
Chronic kidney disease	701 (7.6)	72 (7.5)	734 (11.0)
≥1 Hospitalization	5125 (55.3)	514 (53.8)	3794 (56.9)
Antiplatelet drugs	2447 (26.4)	210 (22)	1783 (26.8)
Cardiovascular drugs	5563 (60.0)	518 (54.2)	4377 (65.7)
Covariates included in the multivariate model			
Glucocorticoids	7064 (58.5)	607 (51.3)	4878 (53.1)
SLE treatments (other than HCQ)^a^	2470 (20.5)	246 (20.8)	2501 (27.2)
Nonsteroidal anti-inflammatory drugs	4407 (36.5)	447 (37.8)	3059 (33.3)
Medical visits, mean (SD), No.	10.58 (10.8)	11.13 (13.4)	10.68 (13.4)

Abbreviations: ALD, affection of long duration; CV, cardiovascular; HCQ, hydroxychloroquine; SLE, systemic lupus erythematosus.

^a^ SLE treatments other than HCQ included mycophenolate mofetil, azathioprine, thalidomide, methotrexate, belimumab, and cyclophosphamide.

### Association Between Hydroxychloroquine and CV Events

Current hydroxychloroquine users, compared with patients with no exposure within 365 days prior to the index date, had lower odds of the composite CV outcome (aOR, 0.63; 95% CI, 0.57-0.70). Specifically, odds were lower for MI (aOR, 0.72; 95% CI, 0.60-0.87), stroke (aOR, 0.71; 95% CI, 0.61-0.83), and OTE (aOR, 0.58; 95% CI, 0.48-0.69) (Table 4). Conversely, for remote hydroxychloroquine exposure vs no exposure within the past 365 days, the odds of the composite CV outcome and its individual components were closer to 1 and not statistically significant.

**Table 4.** Association Between Hydroxychloroquine Exposure and Cardiovascular Events in Patients With Systemic Lupus Erythematosus

Outcome	No.		OR (95% CI)	
	Patients with a CV event	Controls	Crude	Adjusted^a^
**CV event**				
No.	1981	16 892	NA	NA
No exposure within 365 d	984	6663	1 [Reference]	1 [Reference]
Remote exposure	130	955	0.91 (0.74-1.11)	0.95 (0.77-1.16)
Current exposure	867	9274	0.63 (0.57-0.69)	0.63 (0.57-0.70)
**Myocardial infarction**				
No.	623	5323	NA	NA
No exposure within 365 d	319	2263	1 [Reference]	1 [Reference]
Remote exposure	34	319	0.79 (0.54-1.16)	0.84 (0.57-1.23)
Current exposure	270	2741	0.72 (0.60-0.85)	0.72 (0.60-0.87)
**Stroke**				
No.	865	7455	NA	NA
No exposure within 365 d	427	3090	1 [Reference]	1 [Reference]
Remote exposure	56	440	0.89 (0.65-1.20)	0.93 (0.68-1.27)
Current exposure	382	3925	0.69 (0.60-0.81)	0.71 (0.61-0.83)
**Thromboembolism**				
No.	664	5662	NA	NA
No exposure within 365 d	336	2230	1 [Reference]	1 [Reference]
Remote exposure	48	365	0.86 (0.62-1.19)	0.87 (0.62-1.21)
Current exposure	280	3067	0.58 (0.49-0.69)	0.58 (0.48-0.69)
**Pulmonary embolism**				
No.	249	2078	NA	NA
No exposure within 365 d	136	850	1 [Reference]	1 [Reference]
Remote exposure	20	136	0.94 (0.56-1.56)	0.91 (0.54-1.53)
Current exposure	93	1092	0.49 (0.37-0.66)	0.48 (0.35-0.65)
**Venous thromboembolism**				
No.	450	3903	NA	NA
No exposure within 365 d	217	1516	1 [Reference]	1 [Reference]
Remote exposure	35	258	0.93 (0.63-1.37)	0.94 (0.64-1.39)
Current exposure	198	2129	0.64 (0.52-0.79)	0.63 (0.51-0.78)

Abbreviations: CV, cardiovascular; NA, not applicable; NSAIDs, nonsteroidal anti-inflammatory drugs; OR, odds ratio; SLE, systemic lupus erythematosus.

^a^ Adjusted for the prior use of glucocorticoids, SLE treatments (other than hydroxychloroquine), NSAIDs in the 365 days before the index date, medical visits in the 365 days before the index date, as well as for the history of other CV events of interest (for the analysis of myocardial infarction, stroke, and other thromboembolic events).

Sensitivity analyses stratified by age, controlling for additional covariates, in patients allowed 2 years before registry entry, among patients with a previous CV event, in patients with and without history of CV disease, and, stratified by presence of APLS showed consistent protective associations of current hydroxychloroquine exposure and remained significant for the composite CV event (eTables 5-10 in Supplement 1). Adding 30 days to the duration of exposure for the final delivery of hydroxychloroquine and allowing up to 120 days per dispensing for same-day deliveries, yielded the same odds of the composite CV outcome (aOR, 0.64 [95% CI, 0.58-0.71]) for current hydroxychloroquine users. Among patients with a CV event, 1034 patients (52.2%) had prior exposure to hydroxychloroquine within the 4 years preceding the 365 days before the index date (past exposure), compared with 9703 patients (57.4%) among controls (eTable 11 in Supplement 1). These proportions were 273 patients (27.7%) and 2090 patients (31.4%) for patients with a CV event and controls categorized as not exposed within 365 days, respectively. Adjusting the primary analysis for past hydroxychloroquine exposure found lower odds of the composite CV outcome for current hydroxychloroquine exposure (aOR, 0.65; 95% CI, 0.58-0.73). The findings for past hydroxychloroquine exposure were not statistically significant (aOR, 0.97; 95% CI, 0.79-1.20).

## Discussion

In this cohort study, we observed that current use of hydroxychloroquine was associated with a reduced risk of MI, stroke, OTEs, and the composite CV outcome in both primary and sensitivity analyses. This protective association was consistent even among patients with a history of CV disease, those with and without APLS, and across all age groups, with age-dependent variations in hydroxychloroquine’s protective associations against specific CV events. Remote hydroxychloroquine exposure was not associated with a reduced risk of these CV events.

One crucial observation was that the characteristics of current hydroxychloroquine users did not differ significantly from those of patients who discontinued treatment 91 to 365 days prior to a CV event. This finding may reflect the mechanism of action of hydroxychloroquine during thrombotic events. Recent evidence suggests that immune cells play a key role in the onset of CV diseases.^14,15,16^ The association between autoimmune diseases and a higher risk of CV complications has been well established, including in a 2022 population-based study in the United Kingdom.^17^ Therefore, inflammation is considered a risk factor for the development of CV events. The pathways leading to endothelial dysfunction and vascular damage have become a field of interest, especially in identifying new targets for drug therapy.^18^ Several studies have described the effects of hydroxychloroquine on endothelial dysfunction.^19,20^ However, the impact of ongoing use (vs past use) on inflammation and CV disease remains unclear.

Despite improvements in patient survival, patients with SLE have an increased risk of accelerated atherosclerosis^17^ facilitated by steroids, antiphospholipid antibodies, and SLE activity itself. Following the European Alliance of Associations for Rheumatology recommendations,^4^ patients with SLE should be treated with hydroxychloroquine continuously, as they have a better overall relative survival rate and a lower risk of developing severe SLE and organ damage.^5,7^ The discontinuation of hydroxychloroquine may increase the risk of disease activity.^5^

Hydroxychloroquine is beneficial for the treatment of CV diseases.^21,22,23,24^ A recent nested case-control study of 10 268 patients with incident CV events and 29 969 controls conducted using British Columbia, Canada, administrative health databases found that patients with SLE or rheumatoid arthritis currently using hydroxychloroquine had a lower risk of thrombotic events.^8^ However, that study did not reach statistical significance for any CV events in the SLE subcohort.

Our findings regarding the risk of OTE are consistent with those of other observational studies.^25,26,27^ However, to our knowledge, this is the first study with usual-care data showing the benefits of hydroxychloroquine in improving arterial outcomes in patients with SLE. This finding is consistent with 2 studies^8,16^ reporting a lower risk of MI in patients with rheumatoid arthritis treated with hydroxychloroquine. Further studies are needed to understand the relationship between CV disease and hydroxychloroquine. A key strength of the study is its representation of 99% of the French population, providing a vast pool of potential controls and enabling robust matching and adjustment for numerous potential confounders.

### Limitations

This study has some limitations. Like most observational studies, there is particular concern regarding potential confounding by indication. In our analysis, current hydroxychloroquine users were significantly younger than nonusers, with a difference of nearly 5 years in mean age. More importantly, for current hydroxychloroquine users, the duration since the onset of SLE was significantly (2-fold) longer than that for hydroxychloroquine nonusers. Additionally, they had a higher prevalence of CKD. However, the matching process effectively balanced these variables between the patients with CV events and controls; additional adjustments for age aimed to minimize residual confounding related to this factor. Nevertheless, residual confounding may persist due to unmeasured risk factors for CV events. An inherent limitation of the SNDS is the lack of clinical and biological data, which could have provided deeper insights into the distinctions between current users and nonusers of hydroxychloroquine. Furthermore, the reasons why patients discontinued hydroxychloroquine were not documented, limiting our understanding of treatment patterns, and their potential impact on study outcomes.

Another limitation is the accuracy of diagnosis, both in defining study populations and assessing outcomes. Specific studies validating the SNDS for SLE were not identified, but the inclusion of stringent criteria and a mean follow-up period of more than 10 years aimed to mitigate potential misclassification. Additionally, the SNDS has shown a high positive predictive value for CV outcomes, particularly those examined in this study, enhancing the reliability of our findings. Any diagnostic inaccuracies would bias results toward the null hypothesis.

Another limitation is the absence of direct information on treatment adherence. Patients acquiring medications during the remote exposure period may not have strictly adhered to treatment, potentially influencing their actual exposure during the current time window. However, adjusting for a grace period of 30 days to the last exposure did not alter our results.

Another limitation is the absence of race and ethnicity data in the SNDS. However, there are no differences in access to medication according to race or ethnicity in France, as universal medication and health services coverage are available. Furthermore, the reduced representation of older male in the matched sample limits the generalizability of our findings to this demographic.

## Conclusions

In this cohort study using a nested case-control design and a national health care database, current treatment with hydroxychloroquine in patients with SLE was associated with a significantly reduced risk of MI, stroke, and OTEs compared with untreated patients. This protective association was not observed after discontinuation of hydroxychloroquine treatment. These findings support the protective association of hydroxychloroquine against CV events and underscore the importance of continuous hydroxychloroquine therapy for patients diagnosed with SLE.

## Notes

Supplement 1. **eTable 1.** Definitions and Codes for the Identification of the Cohort and the Case Patients **eTable 2.** Definitions and Codes for the Matching Variables **eTable 3.** Codes for the Covariates Included in the Multivariate Model **eTable 4.** Descriptive Analyses for Cases of Each CV Event **eTable 5.** Association Between Hydroxychloroquine Exposure and Cardiovascular Events in the Cohort of Systemic Lupus Erythematosus Patients by Class of Age **eTable 6.** Association Between Hydroxychloroquine Exposure and Cardiovascular Events in the Cohort of Systemic Lupus Erythematosus Patients When Adjusted for Current Use of Antiplatelet and Cardiovascular Drugs **eTable 7.** Association Between Hydroxychloroquine Exposure and Cardiovascular Events in the Cohort of Patients With Systemic Lupus Erythematosus Allowing Only for a 2-Year Delay Before Entry in the Study Population (Instead of 5 Years in the Primary Analysis) **eTable 8.** Association Between Hydroxychloroquine Exposure and Cardiovascular Events in the Cohort of Patients With Systemic Lupus Erythematosus With a Previous Cardiovascular Outcome **eTable 9.** Association Between Hydroxychloroquine Exposure and Cardiovascular Events in the Cohort of Patients With Systemic Lupus Erythematosus by Use of Antiaggregant and Cardiovascular Drugs **eTable 10.** Association Between Hydroxychloroquine Exposure and Cardiovascular Events in the Cohort of Patients With Systemic Lupus Erythematosus by the Presence of Antiphospholipid Syndrome **eTable 11.** Association Between Hydroxychloroquine Exposure and Cardiovascular Events in the Cohort of Patients With Systemic Lupus Erythematosus Controlled for Past Exposure to Hydroxychloroquine

Supplement 2.
Data Sharing Statement
