# Supplementary material for: Hydroxychloroquine and Cardiovascular Events in Patients With Systemic Lupus Erythematosus
Source: JAMA Netw Open. 2024 Aug 30;7(8):e2432190. doi: 10.1001/jamanetworkopen.2024.32190 (PMC11364994; doi:10.1001/jamanetworkopen.2024.32190)
Supplement: Supplement 1. — eTable 1. Definitions and Codes for the Identification of the Cohort and the Case Patients eTable 2. Definitions and Codes for the Matching Variables eTable 3. Codes for the Covariates Included in the Multivariate Model eTable 4. Descriptive Analyses for Cases of Each CV Event eTable 5. Association Between Hydroxychloroquine Exposure and Cardiovascular Events in the Cohort of Systemic Lupus Erythematosus Patients by Class of Age eTable 6. Association Between Hydroxychloroquine Exposure and Cardiovascular Events in the Cohort of Systemic Lupus Erythematosus Patients When Adjusted for Current Use of Antiplatelet and Cardiovascular Drugs eTable 7. Association Between Hydroxychloroquine Exposure and Cardiovascular Events in the Cohort of Patients With Systemic Lupus Erythematosus Allowing Only for a 2-Year Delay Before Entry in the Study Population (Instead of 5 Years in the Primary Analysis) eTable 8. Association Between Hydroxychloroquine Exposure and Cardiovascular Events in the Cohort of Patients With Systemic Lupus Erythematosus With a Previous Cardiovascular Outcome eTable 9. Association Between Hydroxychloroquine Exposure and Cardiovascular Events in the Cohort of Patients With Systemic Lupus Erythematosus by Use of Antiaggregant and Cardiovascular Drugs eTable 10. Association Between Hydroxychloroquine Exposure and Cardiovascular Events in the Cohort of Patients With Systemic Lupus Erythematosus by the Presence of Antiphospholipid Syndrome eTable 11. Association Between Hydroxychloroquine Exposure and Cardiovascular Events in the Cohort of Patients With Systemic Lupus Erythematosus Controlled for Past Exposure to Hydroxychloroquine [file jamanetwopen-e2432190-s001.pdf]

Supplemental Online Content

Grimaldi-Bensouda L, Duchemin T, Hamon Y, et al. Hydroxychloroquine and cardiovascular events in patients with systemic lupus erythematosus. *JAMA Netw Open*. 2024;7(8):e2432190. doi:10.1001/jamanetworkopen.2024.32190

eTable 1. Definitions and Codes for the Identification of the Cohort and the Case Patients
eTable 2. Definitions and Codes for the Matching Variables
eTable 3. Codes for the Covariates Included in the Multivariate Model
eTable 4. Descriptive Analyses for Cases of Each CV Event
eTable 5. Association Between Hydroxychloroquine Exposure and Cardiovascular Events in the Cohort of Systemic Lupus Erythematosus Patients by Class of Age
eTable 6. Association Between Hydroxychloroquine Exposure and Cardiovascular Events in the Cohort of Systemic Lupus Erythematosus Patients When Adjusted for Current Use of Antiplatelet and Cardiovascular Drugs
eTable 7. Association Between Hydroxychloroquine Exposure and Cardiovascular Events in the Cohort of Patients With Systemic Lupus Erythematosus Allowing Only for a 2-Year Delay Before Entry in the Study Population (Instead of 5 Years in the Primary Analysis)
eTable 8. Association Between Hydroxychloroquine Exposure and Cardiovascular Events in the Cohort of Patients With Systemic Lupus Erythematosus With a Previous Cardiovascular Outcome
eTable 9. Association Between Hydroxychloroquine Exposure and Cardiovascular Events in the Cohort of Patients With Systemic Lupus Erythematosus by Use of Antiaggregant and Cardiovascular Drugs
eTable 10. Association Between Hydroxychloroquine Exposure and Cardiovascular Events in the Cohort of Patients With Systemic Lupus Erythematosus by the Presence of Antiphospholipid Syndrome
eTable 11. Association Between Hydroxychloroquine Exposure and Cardiovascular Events in the Cohort of Patients With Systemic Lupus Erythematosus Controlled for Past Exposure to Hydroxychloroquine

This supplemental material has been provided by the authors to give readers additional information about their work.

© 2024 Grimaldi-Bensouda L et al. *JAMA Network Open.*

eTable 1. Definitions and Codes for the Identification of the Cohort and Case Patients

Definition	***Code***
Disseminated systemic lupus erythematosus with organ damage	ICD-10 code: M321
Other types of disseminated systemic lupus erythematosus	ICD-10 code: M328
Disseminated systemic lupus erythematosus without other information	ICD-10 code: M329
Mycophenolate mofetil	ATC: L04AA06
Azathioprine	ATC: L04AX01
Hydroxychloroquine	ATC: P01BA02
Thalidomide	ATC: L04AX02
Methotrexate	ATC: L04AX03
Belimumab	ATC: L04AA26
Cyclophosphamide	ATC: L01AA01
Myocardial Infarction	ICD-10 code: I21
Stroke	ICD-10 code: G45, I63, I64
Phlebitis and thrombophlebitis	ICD-10 code: I80
Other venous thrombosis and embolism	ICD-10 code: I829
Venous thromboembolism and pulmonary embolism	ICD-10 code: I26

Abbreviations: ATC, Anatomical Therapeutic Classification; ICD, International Classification of Diseases

© 2024 Grimaldi-Bensouda L et al. *JAMA Network Open.*

eTable 2. Definitions and Codes for the Matching Variables

Definition	***Code***
Lupus ALD code	ICD-10 code: M32
Platelet aggregation inhibitors drugs	ATC codes beginning with B01AC
Cardiovascular drugs	ATC codes beginning with C
Chronic kidney disease	ICD-10 code: N18

Abbreviations: ALD, Affection of Long Duration; ATC, Anatomical Therapeutic Classification; ICD, International Classification of Diseases.

© 2024 Grimaldi-Bensouda L et al. *JAMA Network Open.*

eTable 3. Codes for the Covariates Included in the Multivariate Model

Covariate	***Code***
Nonsteroidal anti-inflammatory drugs	ATC codes beginning with M01
Glucocorticoids	ATC codes beginning with H02AB
Other SLE treatments (other than HCQ)	ATC codes<br>J04BA02–Dapsone<br>J06BA01–Immunoglobulin and hyaluronidase<br>J06BA02–Immunoglobulins, normal human, for intravascular adm.<br>L01AA01–Cyclophosphamide<br>L01XC02–Rituximab<br>L04AA06–Mycophenolic Mofetil<br>L04AA24–Abatacept<br>L04AA26–Belimumab<br>L04AD01–Ciclosporine<br>L04AD02–Tacrolimus<br>L04AX01 – Azathioprine<br>L04AX03–Methotrexate<br>P01BA01–Chloroquine
Cancer	ICD-10 codes: C00 C01 C02 C03 C04 C05 C06 C07 C08 C09 C10 C11 C12 C13 C14 C15 C16 C17 C18 C19 C20 C21 C22 C23 C24 C25 C26 C30 C31 C32 C33 C34 C35 C36 C37 C38 C39 C40 C41 C43 C44

© 2024 Grimaldi-Bensouda L et al. *JAMA Network Open.*

	C45 C46 C47 C48 C49 C50 C51 C52 C53 C54 C55 C56 C57 C58 C60 C61 C62 C63 C64 C65 C66 C67 C68 C69 C70 C71 C72 C73 C74 C75 C76 C78 C79 C80 D00 D01 D02 D03 D04 D05 D06 D07 D09<br>C77 Z850 Z851 Z852 Z853 Z854 Z855 Z858 Z511<br>Z856 Z857 Z862 Z85 C81 C82 C83 C84 C85 C86 C88 C90 C91 C92 C93 C94 C95 C96 Z511<br>CCAM code: EBLA003 FCFA022 FCFC004 FCFA025 FCFA019 FCFA010 FCFA011 FCFA029 FCFC003 FCFC001 FCFA006 FCFC005 FCFA002 FCFA004 FCFA009 FCFA005 FCFA016 FCFA027 FCFA008 FCFA013 FCFA020 YYYY026 YYYY031 ZZNL045 ZZNL049 ZZNL050 ZZNL048 ZZNL047 ZZNL046 ZANL001 ZZNL051 ZZNL052 KCNL004 PANL001 AZNL001 KCNL003 ZZNL055 ZZNL058 ZZNL053 ZZNL054 ZZNL060 ZZNL059 ZZNL061 ZZNL066 ZZNL064 ZZNL065 ZZNL063 ZZNL062 YYYY493 YYYY511 YYYY520 YYYY500 YYYY497 YYYY522 YYYY533 YYYY577 YYYY588 YYYY566 YYYY555 YYYY544 YYYY599 YYYY492 YYYY470

© 2024 Grimaldi-Bensouda L et al. *JAMA Network Open.*

	YYYY299 YYYY267 YYYY301 YYYY302 YYYY303 YYYY256 YYYY244 YYYY175 YYYY197 YYYY211 YYYY223 YYYY304 YYYY305 YYYY316 YYYY315 YYYY320 YYYY323 YYYY324 YYYY314 YYYY313 YYYY306 YYYY307 YYYY310 YYYY312 YYYY152 YYYY141 YYYY045 YYYY023 YYYY046 YYYY047 YYYY048 YYYY049 YYYY050 YYYY099 YYYY101 YYYY122 YYYY136 YYYY056 YYYY055 YYYY051 YYYY052 YYYY053 YYYY054 YYYY325 YYYY326 YYYY391 YYYY390 YYYY392 YYYY393 YYYY398 YYYY387 YYYY383 YYYY379 YYYY380 YYYY381 YYYY382 YYYY450 YYYY451 YYYY471 YYYY479 YYYY480 YYYY481 YYYY469 YYYY468 YYYY457 YYYY458 YYYY459 YYYY460 YYYY377 YYYY371 YYYY343 YYYY338 YYYY345 YYYY346 YYYY347 YYYY337 YYYY336 YYYY327 YYYY331 YYYY334 YYYY335 YYYY348 YYYY349 YYYY367 YYYY368 YYYY369 YYYY370 YYYY365 YYYY360 YYYY356 YYYY357 YYYY358 YYYY359 YYYY491 ZZNL002 ZZNL003 ZZNL004 ZZNL001 ZZNL015 JLNL008

© 2024 Grimaldi-Bensouda L et al. *JAMA Network Open.*

	BHNL001 ZZNL005 ZZNL006 ZZNL011<br>ZZNL012 ZZNL013 ZZNL010 ZZNL009<br>ZZNL007 ZZNL008 JLNL007 JLNL006<br>JKNL001 JKNL002 JKNL003 JGNL001<br>ZZNL017 ZZNL019 ZZNL018 JKNL004<br>JKNL005 JLNL004 JLNL005 JLNL003 JLNL002<br>JKNL006 JLNL001 ZZNL014 JDLD659
Antiphospholipid syndrome	ICD-10 codes: D686 and D688
Lupus nephritis	ICD-10 codes beginning with N0 or N1
Diabetes	ATC codes: A10<br>ICD-10 codes: E10 E11 E12 E13 E14 G590 G632<br>G990 H280 H36 M142 N083<br>LPP codes (medical device): 1101720 6187287<br>6186260 6111769 6113395 6115508 6112898<br>6112154 6112190 6124341 6126819 6131594<br>6177656 6178963 6185495 6184633 6111380<br>1103570 1102257 1110720 1190296 1167498<br>1173487 6166109 1136894 6188051 6186224<br>6113426 6115520 6112912 6166115 6116637<br>6126920 6174758 6185420 6185443 6179595<br>6184679 6110818 6111350 1186722 6187264<br>6187590 6111752 6113461 6112639 6162459<br>6112183 6124602 6126937 6131588 6177691<br>6185414 6184662 6185093 6110089 1180441

© 2024 Grimaldi-Bensouda L et al. *JAMA Network Open.*

	1187408 6111730 1198033 6187270 6188068 6186230 6110149 6113490 6115566 6162465 6112125 6169310 6112250 6124594 6126890 6131602 6177640 6185450 6185466 6184640 6185124 6110824 6110072 6110563 6111367 1172861 1108350 6174735 1126737 1145999 1155302 1186202 1170862 1164991 1163856 1121705 1117454 6188499 6186276 6186282 6111723 6113403 6115514 6112964 6162436 6112214 6166121 6112220 6124625 6126966 6134629 6177662 6185503 6185510 6184685 6110847 6110103 6111396 1180665 6186247 1141412 6169303 6148130 6126972 6110830 1101826 6112935 6166138 6126989 1198145 1166100 6187525 6187293 6186253 6111717 6113455 6115550 6162442 6112243 6166150 6116620 6148169 6124619 6134612 6174741 6177679 6185472 6184691 6185087 6185213 6113478 6150226 6111373 1138090 1128334 1186774 6177685 6188074 6185526 6185101 1147308 1142883 6188080 6186299 6113449 6115537 6113219 6112237 6124631 6131619 6185532 6185118 6185207 1199297

Abbreviations: Adm., administration; ATC, Anatomical Therapeutic Classification; HCQ, hydroxychloroquine; SLE, systemic lupus erythematosus

© 2024 Grimaldi-Bensouda L et al. *JAMA Network Open.*

eTable 4. Descriptive Analyses for Cases of Each Cardiovascular Event (MI, Stroke, or TEE Separately)

	**Case**	**Control**
**Myocardial infarction**		
n	623	5,323
Age in years, mean (SD)	61.4 (14.15)	61.39 (14.14)
Time since onset of illness in year, mean (SD)	14.24 (7.67)	12.47 (7.31)
Sex, n (%)		
Female	449 (72.1%)	3,836 (72.1%)
Male	174 (27.9%)	1,487 (27.9%)
Chronic kidney disease, n (%)	92 (14.8%)	786 (14.8%)
At least one hospitalisation, n (%)	344 (55.2%)	2,939 (55.2%)
Antiplatelet drugs n (%)	238 (38.2%)	2,034 (38.2%)
Cardiovascular drugs, n (%)	455 (73%)	3,888 (73%)
Glucocorticoids, n (%)	385 (61.8%)	3,037 (57.1%)
Lupus drugs (other than HCQ), n (%)	150 (24.1%)	1,368 (25.7%)
NSAIDs, n (%)	183 (29.4%)	1,737 (32.6%)
Consultations (n)	11.02 (13.09)	10.68 (11.73)
**Stroke / TIA**		
n	865	7,455
Age in years, mean (SD)	59.62 (16.69)	59.61 (16.7)

© 2024 Grimaldi-Bensouda L et al. *JAMA Network Open.*

	**Case**	**Control**
Time since onset of illness in year, mean (SD)	13.22 (7.79)	11.9 (7.21)
Sex, n (%)		
Female	727 (84%)	6,266 (84%)
Male	138 (16%)	1,189 (16%)
Chronic kidney disease, n (%)	106 (12.3%)	914 (12.3%)
At least one hospitalisation, n (%)	472 (54.6%)	4,068 (54.6%)
Antiplatelet drugs n (%)	258 (29.8%)	2,224 (29.8%)
Cardiovascular drugs, n (%)	564 (65.2%)	4,861 (65.2%)
Glucocorticoids, n (%)	511 (59.1%)	4,230 (56.7%)
Lupus drugs (other than HCQ), n (%)	201 (23.2%)	1,692 (22.7%)
NSAIDs, n (%)	283 (32.7%)	2,617 (35.1%)
Consultations (n)	11.01 (10.1)	10.57 (11.34)
**Thromboembolism**		
n	664	5,662
Age in years, mean (SD)	53.38 (17.08)	53.38 (17.07)
Time since onset of illness in year, mean (SD)	12.05 (7.52)	10.93 (6.9)
Sex, n (%)		
Female	547 (82.4%)	4,664 (82.4%)
Male	117 (17.6%)	998 (17.6%)
Chronic kidney disease, n (%)	108 (16.3%)	921 (16.3%)
At least one hospitalisation, n (%)	438 (66%)	3,735 (66%)

© 2024 Grimaldi-Bensouda L et al. *JAMA Network Open.*

	Case	Control
Antiplatelet drugs n (%)	170 (25.6%)	1,450 (25.6%)
Cardiovascular drugs, n (%)	374 (56.3%)	3,189 (56.3%)
Glucocorticoids, n (%)	469 (70.6%)	3,404 (60.1%)
Lupus drugs (other than HCQ), n (%)	205 (30.9%)	1,658 (29.3%)
NSAIDs, n (%)	229 (34.5%)	1,978 (34.9%)
Consultations (n)	13.86 (18.31)	11.4 (12.79)
**Pulmonary embolism**		
n	249	2,078
Age in years, mean (SD)	53.26 (16.38)	53.27 (16.36)
Time since onset of illness in year, mean (SD)	12.89 (7.85)	11.91 (7.26)
Sex, n (%)		
Female	197 (79.1%)	1,644 (79.1%)
Male	52 (20.9%)	434 (20.9%)
Chronic kidney disease, n (%)	66 (26.5%)	551 (26.5%)
At least one hospitalisation, n (%)	172 (69.1%)	1,435 (69.1%)
Antiplatelet drugs n (%)	62 (24.9%)	517 (24.9%)
Aspirin >100mg, n (%)	52 (20.9%)	439 (21.1%)
Glucocorticoids, n (%)	180 (72.3%)	1,289 (62%)
Lupus drugs (other than HCQ), n (%)	0 (0%)	0 (0%)
NSAIDs, n (%)	81 (32.5%)	652 (31.4%)
Consultations (n)	17.41 (25.99)	12.22 (14.98)
**Venous thromboembolism**		

© 2024 Grimaldi-Bensouda L et al. *JAMA Network Open.*

	**Case**	**Control**
n	450	3,903
Age in years, mean (SD)	53.4 (17.48)	53.4 (17.47)
Time since onset of illness in year, mean (SD)	11.54 (7.33)	10.34 (6.64)
Sex, n (%)		
Female	379 (84.2%)	3,287 (84.2%)
Male	71 (15.8%)	616 (15.8%)
Chronic kidney disease, n (%)	47 (10.4%)	408 (10.4%)
At least one hospitalisation, n (%)	293 (65.1%)	2,541 (65.1%)
Antiplatelet drugs n (%)	117 (26%)	1,015 (26%)
Cardiovascular drugs, n (%)	250 (55.6%)	2,168 (55.6%)
Glucocorticoids, n (%)	313 (69.6%)	2,306 (59.1%)
Lupus drugs (other than HCQ), n (%)	127 (28.2%)	1,063 (27.2%)
NSAIDs, n (%)	161 (35.8%)	1,435 (36.8%)
Consultations (n)	11.72 (10.81)	10.96 (11.57)

Abbreviations: ALD, Affection of Long Duration; HCQ, hydroxychloroquine; NSAID, nonsteroidal anti-inflammatory drug; SLE, systemic lupus erythematosus.

© 2024 Grimaldi-Bensouda L et al. *JAMA Network Open.*

eTable 5. Association between Hydroxychloroquine Exposure and Cardiovascular Events in the Cohort of Patients with Systemic Lupus Erythematosus by Class of Age

	Cases	Controls	Crude ORs [95% CI]	aORs [95% CI]
**Patients with SLE (<59 years)**				
**CV event**	**1,012**	**8,930**		
No exposure	438	3,032	Reference	Reference
Remote exposure	71	548	0.88 [0.67–1.15]	0.91 [0.69–1.20]
Current exposure	503	5,350	0.63 [0.55–0.73]	0.65 [0.56–0.75]
**Myocardial infarction**	**269**	**2,378**		
No exposure	133	853	Reference	Reference
Remote exposure	18	152	0.86 [0.51–1.44]	0.9 [0.53–1.54]
Current exposure	118	1,373	0.55 [0.42–0.72]	0.56 [0.42–0.74]
**Stroke/TIA**	**405**	**3,582**		
No exposure	145	1,247	Reference	Reference
Remote exposure	30	222	1.06 [0.69–1.63]	1.15 [0.74–1.77]
Current exposure	230	2,113	0.92 [0.73–1.15]	0.95 [0.76–1.20]
**Thromboembolism**	**407**	**3,559**		
No exposure	193	1,246	Reference	Reference
Remote exposure	27	253	0.67 [0.44–1.03]	0.66 [0.43–1.02]
Current exposure	187	2,060	0.54 [0.44–0.68]	0.54 [0.43–0.68]
**Pulmonary embolism**	**152**	**1,252**		
No exposure	77	463	Reference	Reference
Remote exposure	10	92	0.71 [0.35–1.43]	0.68 [0.33–1.39]

© 2024 Grimaldi-Bensouda L et al. *JAMA Network Open.*

Current exposure	65	697	0.5 [0.35–0.72]	0.49 [0.34–0.71]
**Venous thromboembolism**	**277**	**2,512**		
No exposure	126	858	Reference	Reference
Remote exposure	22	179	0.79 [0.49–1.29]	0.78 [0.48–1.28]
Current exposure	129	1,475	0.56 [0.43–0.73]	0.56 [0.42–0.73]
**Patients with SLE (≥59 years)**				
**Thrombotic event (composite)**	**969**	**7,962**		
No exposure	546	3631	Reference	Reference
Remote exposure	59	407	0.95 [0.71–1.27]	0.99 [0.74–1.34]
Current exposure	364	3924	0.62 [0.53–0.71]	0.63 [0.54–0.73]
**Myocardial infarction**	**354**	**2,945**		
No exposure	186	1,410	Reference	Reference
Remote exposure	16	167	0.71 [0.41–1.22]	0.75 [0.43–1.30]
Current exposure	152	1,368	0.88 [0.69–1.11]	0.89 [0.70–1.14]
**Stroke/TIA**	**460**	**3,873**		
No exposure	282	1,843	Reference	Reference
Remote exposure	26	218	0.79 [0.52–1.22]	0.79 [0.51–1.23]
Current exposure	152	1,812	0.55 [0.44–0.67]	0.55 [0.44–0.68]
**Thromboembolism**	**257**	**2,103**		
No exposure	143	984	Reference	Reference
Remote exposure	21	112	1.27 [0.77–2.11]	1.36 [0.81–2.29]
Current exposure	93	1,007	0.64 [0.48–0.85]	0.64 [0.48–0.85]

© 2024 Grimaldi-Bensouda L et al. *JAMA Network Open.*

**Pulmonary embolism**	**97**	**826**		
No exposure	59	387	Reference	Reference
Remote exposure	10	44	1.4 [0.66–3.01]	1.35 [0.61–2.99]
Current exposure	28	395	0.47 [0.29–0.76]	0.44 [0.27–0.73]
**Venous thromboembolism**	**173**	**1,391**		
No exposure	91	658	Reference	Reference
Remote exposure	13	79	1.22 [0.65–2.30]	1.32 [0.69–2.54]
Current exposure	69	654	0.79 [0.56–1.11]	0.79 [0.56–1.12]

* Adjusted for the prior use of glucocorticoids, SLE treatments (other than HCQ), NSAIDs in the 365 days before the index date, medical doctor visits in the 365 days before the index date, as well as for the history of other CV events of interest (for the analysis of MI, stroke, and OTE).

Abbreviations: CI, confidence interval; CV, cardiovascular; MI, myocardial infarction; OR, odds ratio; Ref., reference; SLE, systemic lupus erythematosus

© 2024 Grimaldi-Bensouda L et al. *JAMA Network Open.*

eTable 6: Association between Hydroxychloroquine Exposure and Cardiovascular Events in the Cohort of Patients with Systemic Lupus Erythematosus Controlling for Additional Covariates (Cancer, Diabetes, Lupus Nephritis, Antiphospholipid Syndrome)

	Cases	Controls	Crude ORs [95% CI]	aORs* [95% CI]
**CV event**	**1,981**	**16,892**		
No exposure	984	6,663	Ref.	Ref.
Remote exposure	130	955	0.91 [0.74–1.11]	0.96 [0.79–1.18]
Current exposure	867	9,274	0.63 [0.57–0.69]	0.64 [0.57–0.70]
**Myocardial infarction**	**623**	**5,323**		
No exposure	319	2263	Ref.	Ref.
Remote exposure	34	319	0.79 [0.54 -1.16]	0.84 [0.57–1.24]
Current exposure	270	2,741	0.72 [0.60–0.85]	0.73 [0.61–0.87]
**Stroke/TIA**	**865**	**7,455**		
No exposure	427	3,090	Ref.	Ref.
Remote exposure	56	440	0.89 [0.65–1.20]	0.94 [0.69–1.28]
Current exposure	382	3,925	0.69 [0.60–0.81]	0.71 [0.60–0.83]
**Thromboembolism**	**664**	**5,662**		
No exposure	336	2,230	Ref.	Ref.
Remote exposure	48	365	0.86 [0.62–1.19]	0.88 [0.63–1.23]
Current exposure	280	3,067	0.58 [0.49–0.69]	0.57 [0.48–0.69]
**Pulmonary embolism**	**249**	**2,078**		
No exposure	136	850	Ref.	Ref.

© 2024 Grimaldi-Bensouda L et al. *JAMA Network Open.*

Remote exposure	20	136	0.94 [0.56–1.56]	0.91 [0.54–1.54]
Current exposure	93	1,092	0.49 [0.37–0.66]	0.47 [0.35–0.64]
**Venous thromboembolism**	**450**	**3,903**		
No exposure	217	1516	Ref.	Ref.
Remote exposure	35	258	0.93 [0.63–1.37]	1.04 [0.31–3.46]
Current exposure	198	2,129	0.64 [0.52–0.79]	0.67 [0.37–1.18]

* Adjusted for the prior use of glucocorticoids, SLE treatments (other than HCQ), NSAIDs in the 365 days before the index date, medical doctor visits in the 365 days before the index date, as well as for the history of other CV events of interest (for the analysis of MI, stroke, and OTE), diabetes, cancer, lupus nephritis, and antiphospholipid syndrome.

Abbreviations: CI, confidence interval; CV, cardiovascular; MI, myocardial infarction; OR, odds ratio; Ref., reference; SLE, systemic lupus erythematosus

© 2024 Grimaldi-Bensouda L et al. *JAMA Network Open.*

eTable 7. Association between Hydroxychloroquine Exposure and Cardiovascular Events in the Cohort of Patients with Systemic Lupus Erythematosus Allowing only for a 2-Year Delay before Entry in the Study Population (Instead of 5 Years in the Primary Analysis)

	Cases	Controls	Crude ORs [95% CI]	Adjusted ORs* [95% CI]
**CV event**	**2,922**	**24,778**		
No exposure	1,474	9,367	Ref.	Ref.
Remote exposure	206	1,370	0.94 [0.80–1.11]	0.97 [0.82–1.14]
Current exposure	1,242	14,041	0.56 [0.51–0.61]	0.57 [0.52–0.62]
**Myocardial infarction**	**852**	**7,266**		
No exposure	431	2,994	Ref.	Ref.
Remote exposure	47	439	0.77 [0.56–1.06]	0.8 [0.58–1.11]
Current exposure	374	3,833	0.69 [0.59–0.80]	0.7 [0.60–0.82]
**Stroke/TIA**	**1,276**	**10,902**		
No exposure	638	4,370	Ref.	Ref.
Remote exposure	95	626	1.02 [0.80–1.29]	1.06 [0.83–1.34]
Current exposure	543	5,906	0.62 [0.55–0.71]	0.63 [0.55–0.71]
**Thromboembolism**	**1,008**	**8,494**		
No exposure	526	3,365	Ref.	Ref.
Remote exposure	74	556	0.82 [0.63–1.07]	0.84 [0.64–1.09]
Current exposure	408	4,573	0.55 [0.48–0.64]	0.55 [0.48–0.64]
**Pulmonary embolism**	**418**	**3,430**		

© 2024 Grimaldi-Bensouda L et al. *JAMA Network Open.*

No exposure	222	1,433	Ref.	Ref.
Remote exposure	35	227	1.01 [0.68–1.49]	1 [0.67–1.48]
Current exposure	161	1,770	0.57 [0.45–0.71]	0.56 [0.44–0.70]
**Venous thromboembolism**	**640**	**5,527**		
No exposure	328	2,191	Ref.	Ref.
Remote exposure	48	380	0.79 [0.57–1.11]	0.82 [0.59–1.15]
Current exposure	264	2,956	0.58 [0.48–0.69]	0.58 [0.48–0.69]

* Adjusted for the prior use of glucocorticoids, SLE treatments (other than HCQ), NSAIDs in the 365 days before the index date, medical doctor visits in the 365 days before the index date, as well as for the history of other CV events of interest (for the analysis of MI, stroke, and OTE).

Abbreviations: CI, confidence interval; CV, cardiovascular; MI, myocardial infarction; OR, odds ratio; Ref., reference; SLE, systemic lupus erythematosus

© 2024 Grimaldi-Bensouda L et al. *JAMA Network Open.*

eTable 8. Association between Hydroxychloroquine Exposure and Cardiovascular Events in the Cohort of Patients with Systemic Lupus Erythematosus with a Previous Cardiovascular Outcome

	Cases	Controls	Crude ORs [95% CI]	aORs* [95% CI]
**CV Event**	**144**	**396**		
No exposure	83	162	Ref.	Ref.
Remote exposure	6	24	0.56 [0.22–1.48]	0.48 [0.18–1.32]
Current exposure	55	210	0.51 [0.34–0.78]	0.49 [0.32–0.75]
**Myocardial infarction**	**19**	**33**		
No exposure	9	16	Ref.	Ref.
Remote exposure	2	0	Not enough patients	Not enough patients
Current exposure	8	17	1.03 [0.33–3.20]	0.90 [0.26–3.06]
**Stroke/TIA**	**35**	**62**		
No exposure	21	30	Ref.	Ref.
Remote exposure	2	2	Not enough patients	1.93 [0.19–19.77]
Current exposure	12	30	0.64 [0.28–1.44]	0.68 [0.27–1.71]
**Thromboembolism**	**20**	**59**		
No exposure	10	17	Ref.	Ref.
Remote exposure	0	4	Not enough patients	Not enough patients
Current exposure	10	38	0.49 [0.15–1.62]	0.48 [0.14–1.64]

* Adjusted for the prior use of glucocorticoids, SLE treatments (other than HCQ), NSAIDs in the 365 days before the index date, medical doctor visits in the 365 days before the index

© 2024 Grimaldi-Bensouda L et al. *JAMA Network Open.*

date, as well as for the history of other CV events of interest (for the analysis of MI, stroke, and OTE),

Abbreviations: CI, confidence interval; CV, cardiovascular; MI, myocardial infarction; OR, odds ratio; Ref., reference; SLE, systemic lupus erythematosus

eTable 9. Association between Hydroxychloroquine Exposure and Cardiovascular Events in the Cohort of Patients with Systemic Lupus Erythematosus by Use of Antiaggregant and Cardiovascular Drugs

	Cases	Controls	Crude ORs [95% CI]	aORs* [95% CI]
**Lupus patient with use of antiaggregants or CV drugs**				
**CV event**	**1,349**	**11,169**		
No exposure	682	4,545	**Ref.**	**Ref.**
Remote exposure	71	560	0.83 [0.63–1.08]	0.87 [0.66–1.14]
Current exposure	596	6,064	0.65 [0.57–0.73]	0.66 [0.59–0.75]
**Myocardial infarction**	**474**	**3,931**		
No exposure	243	1,734	**Ref.**	**Ref.**
Remote exposure	22	198	0.81 [0.51–1.28]	0.83 [0.52–1.34]
Current exposure	209	1,999	0.76 [0.62–0.93]	0.77 [0.63–0.95]
**Stroke/TIA**	**607**	**5,119**		
No exposure	313	2,192	**Ref.**	**Ref.**
Remote exposure	37	280	0.9 [0.62–1.30]	0.94 [0.64–1.36]
Current exposure	257	2,647	0.67 [0.56–0.80]	0.68 [0.56–0.81]
**Thromboembolism**	**410**	**3,411**		
No exposure	206	1,374	**Ref.**	**Ref.**
Remote exposure	19	199	0.62 [0.38–1.03]	0.66 [0.39–1.09]
Current exposure	185	1,838	0.64 [0.51–0.80]	0.65 [0.52–0.82]
**Pulmonary embolism**	**157**	**1,281**		
No exposure	84	549	**Ref.**	**Ref.**

© 2024 Grimaldi-Bensouda L et al. *JAMA Network Open.*

Remote exposure	10	71	0.9 [0.44–1.84]	0.83 [0.40–1.73]
Current exposure	63	661	0.56 [0.39–0.81]	0.55 [0.38–0.81]
**Venous thromboembolism**	**278**	**2,353**		
No exposure	136	924	**Ref.**	**Ref.**
Remote exposure	11	147	0.49 [0.26–0.95]	0.32 [0.06–1.59]
Current exposure	131	1,282	0.68 [0.52–0.88]	0.54 [0.28–1.03]
**Lupus patient with no use of antiaggregants or CV drugs**				
**CV event**	**632**	**5,723**		
No exposure	302	2,118	**Ref.**	**Ref.**
Remote exposure	59	395	1.02 [0.75–1.38]	1.04 [0.77–1.42]
Current exposure	271	3,210	0.59 [0.49–0.70]	0.57 [0.48–0.69]
**Myocardial Infarction**	**149**	**1,392**		
No exposure	76	529	**Ref.**	**Ref.**
Remote exposure	12	121	0.74 [0.39–1.40]	0.82 [0.43–1.57]
Current exposure	61	742	0.59 [0.41–0.85]	0.59 [0.40–0.85]
**Stroke/TIA**	**258**	**2,336**		
No exposure	114	898	**Ref.**	**Ref.**
Remote exposure	19	161	0.87 [0.51–1.48]	0.92 [0.54–1.58]
Current exposure	125	1,277	0.76 [0.58–1.00]	0.78 [0.59–1.04]
**Thromboembolism**	**254**	**2,251**		
No exposure	130	856	**Ref.**	**Ref.**
Remote exposure	29	166	1.13 [0.73–1.76]	1.08 [0.69–1.69]

© 2024 Grimaldi-Bensouda L et al. *JAMA Network Open.*

Current exposure	95	1,229	0.5 [0.37–0.66]	0.47 [0.35–0.63]
**Pulmonary embolism**	**92**	**797**		
No exposure	52	301	**Ref.**	**Ref.**
Remote exposure	10	65	0.95 [0.46–1.96]	1.02 [0.49–2.14]
Current exposure	30	431	0.4 [0.24–0.64]	0.37 [0.23–0.61]
**Venous thromboembolism**	**172**	**1,550**		
No exposure	81	592	**Ref.**	**Ref.**
Remote exposure	24	111	1.55 [0.94–2.57]	0.95 [0.20–4.48]
Current exposure	67	847	0.57 [0.40–0.82]	0.59 [0.24–1.46]

* Adjusted for the prior use of glucocorticoids, SLE treatments (other than HCQ), NSAIDs in the 365 days before the index date, medical doctor visits in the 365 days before the index date, as well as for the history of other CV events of interest (for the analysis of MI, stroke, and OTE).

Abbreviations: CI, confidence interval; CV, cardiovascular; MI, myocardial infarction; OR, odds ratio; Ref., reference; SLE, systemic lupus erythematosus

© 2024 Grimaldi-Bensouda L et al. *JAMA Network Open.*

eTable 10: Association between Hydroxychloroquine Exposure and Cardiovascular Events in the Cohort of Patients with Systemic Lupus Erythematosus by the Presence of Antiphospholipid Syndrome.

	Cases	Controls	Crude ORs [95% CI]	aORs* [95% CI]
**Patients with antiphospholipid syndrome**				
**CV event**	**291**	**1,527**		
No exposure	137	483	Ref.	Ref.
Remote exposure	9	75	0.39 [0.18–0.82]	0.40 [0.19–0.86]
Current exposure	145	969	0.55 [0.42–0.73]	0.54 [0.41–0.72]
**Myocardial infarction**	**71**	**339**		
No exposure	34	112	Ref.	Ref.
Remote exposure	2	23	0.42 [0.09–1.91]	0.41 [0.08–1.98]
Current exposure	35	204	0.67 [0.38–1.18]	0.71 [0.39–1.32]
**Stroke/TIA**	**135**	**729**		
No exposure	65	226	Ref.	Ref.
Remote exposure	5	30	0.51 [0.17–1.53]	0.45 [0.15–1.38]
Current exposure	65	473	0.44 [0.29–0.67]	0.43 [0.28–0.66]
**Thromboembolism**	**132**	**730**		
No exposure	63	251	Ref.	Ref.
Remote exposure	4	47	0.31 [0.10–0.91]	0.30 [0.09–0.93]
Current exposure	65	432	0.62 [0.42–0.93]	0.63 [0.41–0.95]
**Pulmonary embolism**	**53**	**277**		
No exposure	25	107	Ref.	Ref.

© 2024 Grimaldi-Bensouda L et al. *JAMA Network Open.*

Remote exposure	2	18	0.32 [0.04–2.66]	0.35 [0.04–2.94]
Current exposure	26	152	0.84 [0.45–1.58]	0.9 [0.47–1.72]
**Venous thromboembolism**	**93**	**535**		
No exposure	42	180	Ref.	Ref.
Remote exposure	5	35	0.54 [0.19–1.50]	0.52 [0.17–1.53]
Current exposure	264	2,956	0.58 [0.36–0.96]	0.56 [0.33–0.93]
**Patients without antiphospholipid syndrome**				
**CV event**	**1,580**	**16,892**		
No exposure	787	6,663	Ref.	Ref.
Remote exposure	112	955	0.97 [0.78–1.21]	1.02 [0.82–1.27]
Current exposure	681	9,274	0.65 [0.58–0.72]	0.66 [0.59–0.74]
**Myocardial Infarction**	**521**	**5,323**		
No exposure	271	2,263	Ref.	Ref.
Remote exposure	29	319	0.8 [0.53–1.20]	0.86 [0.56–1.30]
Current exposure	221	2,741	0.71 [0.59–0.86]	0.73 [0.60–0.89]
**Stroke/TIA**				
No exposure	331	3,090	Ref.	Ref.
Remote exposure	48	441	0.97 [0.70–1.36]	1.04 [0.74–1.45]
Current exposure	303	3,924	0.75 [0.63–0.89]	0.77 [0.65–0.92]
**Thromboembolism**	**487**	**5,662**		
No exposure	249	2,230	Ref.	Ref.
Remote exposure	41	365	1.06 [0.74–1.52]	1.03 [0.71–1.48]
Current exposure	197	3,067	0.58 [0.48–0.72]	0.58 [0.47–0.71]

© 2024 Grimaldi-Bensouda L et al. *JAMA Network Open.*

**Pulmonary Embolism**	**178**	**2,078**		
No exposure	101	850	Ref.	Ref.
Remote exposure	16	136	1.1 [0.62–1.95]	1.02 [0.56–1.87]
Current exposure	61	1,092	0.46 [0.32–0.65]	0.44 [0.30–0.63]
**Venous Thromboembolism**	**331**	**3,903**		
No exposure	162	258	Ref.	Ref.
Remote exposure	29	2,129	1.14 [0.74–1.75]	1.12 [0.72–1.74]
Current exposure	140	1,516	0.64 [0.50–0.82]	0.63 [0.49–0.81]

* Adjusted for the prior use of glucocorticoids, SLE treatments (other than HCQ), NSAIDs in the 365 days before the index date, medical doctor visits in the 365 days before the index date, as well as for the history of other CV events of interest (for the analysis of MI, stroke, and OTE).

Abbreviations: CI, confidence interval; CV, cardiovascular; MI, myocardial infarction; OR, odds ratio; Ref., reference; SLE, systemic lupus erythematosus

© 2024 Grimaldi-Bensouda L et al. *JAMA Network Open.*

eTable 11–Association between Hydroxychloroquine Exposure and Cardiovascular Events in the Cohort of Patients with Systemic Lupus Erythematosus Controlled for 'Past Exposure' to Hydroxychloroquine

	Cases	Controls	aORs* [95% CI]
**CV event**	**1,981**	**16,892**	
No exposure	984	6,663	Ref.
Remote exposure	130	955	0.97 [0.79–1.20]
Current exposure	867	9,274	0.65 [0.58–0.73]
**Myocardial infarction**	**623**	**5,323**	
No exposure	319	2,263	Ref.
Remote exposure	34	319	0.86 [0.58–1.28]
Current exposure	270	2,741	0.74 [0.60–0.92]
**Stroke/TIA**	**865**	**7,455**	
No exposure	427	3,090	Ref.
Remote exposure	56	440	0.99 [0.72–1.36]
Current exposure	382	3,925	0.75 [0.63–0.90]
**Thromboembolism**	**664**	**5,662**	
No exposure	336	2,230	Ref.
Remote exposure	48	365	0.96 [0.67–1.35]
Current exposure	280	3,067	0.63 [0.52–0.78]
**Pulmonary embolism**	**249**	**2,078**	
No exposure	136	850	Ref.
Remote exposure	20	136	0.91 [0.53–1.57]
Current exposure	93	1,092	0.48 [0.34–0.68]

© 2024 Grimaldi-Bensouda L et al. *JAMA Network Open.*

**Venous thromboembolism**	**450**	**3,903**	
No exposure	217	1,516	Ref.
Remote exposure	35	258	1.1 [0.73–1.67]
Current exposure	198	2,129	0.73 [0.57–0.94]

* Adjusted for the prior use of glucocorticoids, SLE treatments (other than HCQ), NSAIDs in the 365 days before the index date, medical doctor visits in the 365 days before the index date, as well as for the history of other CV events of interest (for the analysis of MI, stroke, and OTE).

Abbreviations: CI, confidence interval; CV, cardiovascular; MI, myocardial infarction; OR, odds ratio; Ref., reference; SLE, systemic lupus erythematosus

© 2024 Grimaldi-Bensouda L et al. *JAMA Network Open.*
