# Supplementary material for: Hydroxychloroquine and Cardiovascular Events in Patients With Systemic Lupus Erythematosus
Source: JAMA Netw Open. 2024 Aug 30;7(8):e2432190. doi: 10.1001/jamanetworkopen.2024.32190 (PMC11364994; doi:10.1001/jamanetworkopen.2024.32190)
Supplement: Supplement 2. — Data Sharing Statement [file jamanetwopen-e2432190-s002.pdf]

Data Sharing Statement

Grimaldi-Bensouda. Hydroxychloroquine and Cardiovascular Events in Patients With Systemic Lupus Erythematosus. *JAMA Netw Open*. Published August 30, 2024.
doi:10.1001/jamanetworkopen.2024.32190

Data
Data available: No

Additional Information
Explanation for why data not available: According to data protection and French regulations, the authors cannot publicly release the data from the French National Health Data System (SNDS). However, any person or organisation (public or private; for-profit or non-profit), can access anonymised SNDS data to do a study, research, or an evaluation of public interest, upon authorisation from the French Data Protection Office (See https://www.snds.gouv.fr/SNDS/Processus-d-acces-aux-donnees and https://documentation-snds.health-data-hub.fr/introduction/03-acces-snds.html).
